# Factors Contributing to Retirement Decisions in Denmark: Comparing Employees Who Expect to Retire before, at, and after the State Pension Age

**DOI:** 10.3390/ijerph17093338

**Published:** 2020-05-11

**Authors:** Annette Meng, Emil Sundstrup, Lars L. Andersen

**Affiliations:** National Research Centre for the Working Environment, Lersø Parkallé 105, 2100 Copenhagen, Denmark; ESU@nfa.dk (E.S.); LLA@nfa.dk (L.L.A.)

**Keywords:** senior worker, ageing, occupational health, public health, workplace, sustainable employment

## Abstract

**Aim:** Analyse factors affecting retirement decisions comparing employees expecting to retire before, at, and after the state pension age. **Methods:** In the SeniorWorkingLife cohort, 12,269 workers aged +50 replied to questions about expected retirement age, reasons for leaving, and reasons for staying longer. **Results:** For all groups, poor health, wish for more leisure, and economy were the most salient expected reasons for retiring. Many would stay longer if there were better possibilities for more leisure time, more work flexibility, and economic benefits. Those expecting to retire *before* state pension age were *more* likely to point at desire for more leisure time as expected reason for retiring, and *less* likely to point at economic reasons, and *more* likely to point at health and work demands as possible reasons for prolonging working life. Those expecting to retire *after* state pension age were *more* likely to point at external factors and recognition from the management. **Conclusion:** Factors influencing retirement decisions are similar across the groups. Initiatives for better work–life balance, healthy lifestyle, and economic incentives to continue working may help prevent early retirement and motivate prolonging work life beyond retirement. Results also indicate that less strenuous work is particularly relevant to prevent early retirement.

## 1. Introduction

As a consequence of the ageing population in many western countries, the proportion of older workers and, thus, workers potentially leaving for retirement is increasing. To deal with the potential lack of qualified workers and potential economic strain caused by these demographic changes, there is strong political interest in prolonging work life in most western countries. Thus, knowledge on factors that can prevent early retirement and contribute to prolonged labour market participation is becoming increasingly salient. 

Various factors influence workers’ retirement intentions and behaviour [1,2] and they have been categorised into Push, Pull, Jump, Stuck, and Stay (e.g., Andersen, Jensen [3]). Push refers to factors pushing the worker involuntary out of the labour market such as ill health or adverse working conditions. Pull refers to factors pulling the worker out of the labour market such as economic incentives and Jump to internal motivations to leave the labour market such as the wish for more time for leisure activities. Stay refers to factors motivating the worker to stay at work such as financial gains and work satisfaction and, finally, Stuck to factors leading to the worker being stuck involuntarily at work, for example, because of a poor financial situation so the worker cannot afford to retire. Push, Pull, and Jump are likely to result in early retirement, whereas Stay and Stuck are likely to result in late retirement.

A large body of research on the topic already exists and many factors have been identified influencing workers’ retirement intentions and behaviour. In the present study, we analyse and compare the self-reported salience of central factors underlying Push, Pull, Jump, and Stay among three different retirement groups: workers expecting to retire before, at, or after the state pension age, respectively. Below we provide a brief overview of research on these central factors.

Poor health has been identified as the strongest Push factor. Health status is associated with and predicts time of retirement [4,5,6] and intention to retire early or late [7,8,9]. These findings are confirmed by a meta-analysis showing that poor physical and mental health are important antecedents of early retirement [1]. When looking at qualitative studies on the topic, good health has been described as important or even a precondition for continuing working beyond retirement age [10,11]. Interviews with people aged 65+ who are still working, revealed that own or partners’ ill health was seen as the only threat to continuing working [12]. All in all, ill health appears to be an important Push factor regardless of the timing of the retirement. 

Work demands may likewise push the worker out of the labour market. A scoping review identified physical and cognitive demands at work as barriers for prolonged work participation after pensionable age [2], these findings are supported by another review that showed that physical work demands and work pressure were related to early retirement [13]. Qualitative findings confirm that particularly work pressure [13,14] and to a lesser extent physical work demands [13] contribute to early retirement. On the other hand, a longitudinal study found that psychosocial demands predicted shorter time to preferred retirement but not exit from work per se, and physical work demands did not predict either preferred or actual exit [15]. Furthermore, the meta-analysis by Topa, Depolo [1] found that job stress was a less important antecedent of early retirement. Finally, high work load and physical job demands only marginally increased the risk of early retirement among Danish nurses [7]. Nevertheless, high physical work demand is a strong risk factor for disability pension among Danish healthcare workers [16]. Thus, high physical work demand is a strong Push factor acting through the development of poor health leading to, e.g., disability pension and, thereby, early exit from the labour market. However, it is less clear which role work demands play in regard to early retirement that is not disability pension or for the duration of continuing working beyond retirement age.

Finally, interviews with people who retired early revealed that feeling that their knowledge was not being used in the organisation contributed to pushing them towards early retirement. However, some also reported not wanting to invest in new skills and knowledge and, therefore, retired early to “escape” attending courses and training [14]. 

Other factors motivate workers to leave the labour market rather than pushing them out involuntarily. Research indicates that the decision to retire is influenced by the spouse [8]. Married couples tend to retire at the same time [17] and having a spouse outside the labour market predicts early retirement [7]. Positive attitudes of the spouse with respect to retiring early predict a transition into early retirement [6], and the wish to do enjoyable things with a non-working or older spouse was mentioned as pulling towards early retirement [14]. 

Economy may also work as a Pull factor. Friis, Ekholm [7] found that low income predicted early retirement and argue that this may be because it pays off better to continue working when you have a higher income because the early retirement scheme “income” is relatively low. 

The desire for more leisure time appears to be a strong Jump factor leading older workers towards retirement. Interviewees reported the desire to “enjoy life now”, “wish to do other things outside work”, “wanting more flexibility”, and “to spend more time with or taking care of family members or friends” as motivations for retiring early [14]. A scoping review found that flexible working hours was an incentive for prolonged work life [2], possibly because it facilitates a satisfying work–life balance. Nevertheless, the role of the desire for more leisure as a motivation for retiring after having worked beyond retirement age has, to the authors knowledge, not been explored explicitly.

While the abovementioned factors are likely to lead the worker towards retirement, other factors may contribute to delaying retirement. Although ill health has been identified as a strong Push factor, health considerations can also work as a Stay factor [10,12]. During interviews, older workers described how continuing working keeps you both physically and mentally fit and healthy. Work keeps your mind off pain and health problems, and work obligations and routines help resist “‘giving in’ to illness, laziness, and low mood” [12]. Thus, the desire to keep in good health or take your mind off health problems may contribute to prolonging labour market attachment. 

Furthermore, research indicates that influence and recognition may also work as stay factors motivating employees to continue working. High work time control has been identified as a key factor for extended employment into old age [9]. In addition, decision authority has been found to predict retirement preferences [15], and work autonomy was found to be associated with willingness to work longer [18]. Blekesaune and Solem [19] found low job autonomy to be associated with early retirement among men, but not among women, and argue that this may be because the women are stuck at work due to their financial situation. Finally, low recognition has been found to predict retirement preferences [15] and participants reporting high appreciation at work were less likely to retire early [6]. This is supported by qualitative results indicating that reward and appreciation is important for the motivation to prolong working life [11]. 

Moreover, skill development and challenges at work have been identified as Stay factors. Employees who report greater focus on development of skills and knowledge have been found to be less likely to retire early [6], and challenge at work was found to predict continuing working [11] and willingness to continue working [18]. Armstrong-Stassen and Ursel [20] propose that training and development practices are important for perceived organisational support, which again leads to career satisfaction and ultimately the retention of older workers. These findings are supported by the results of a scoping review that the possibility of up-grading and learning new skills facilitates prolonged work life after retirement. Results from qualitative studies also indicate that the possibility of developing and using one’s skills and knowledge motivates older workers to stay in the work force [10,11,12].

The financial situation can also work as a Stay factor [2]. For example, financial advantages of working have been found to predict continuing working [11], and economic incentives were found to be associated with willingness to work until age 65 or beyond [8]. However, it can also can lead to the person being stuck at the labour market because the person cannot afford to retire at the desired age [4,5,6]. Statements from interviewees in a qualitative study illustrate how the financial situation works both as a Stay and a Stuck factor. Interviewees reported how financial security was motivation for prolonging work life, but in most cases it was to maintain the desired lifestyle more than to provide for basic everyday needs, nevertheless, for some it was necessary to make ends meet [10]. 

Finally, just as having a non-working spouse may pull the worker out of the labour market, qualitative findings show that not wanting to sit home alone while the spouse is working and being single are motivations for continuing work [10].

Lastly, there are strong social norms regarding retirement age and these norms may influence retirement timing [21], this is supported by the finding that workplace timing for retirement is an important antecedent of early retirement [1]. 

As documented in the literature, the retirement decision is often complex and influenced by many factors pulling towards either early or delayed retirement. However, less is known about differences and similarities in regard to Push, Pull, Jump, and Stay factors between workers who intend to leave the labour market before and after the state pension age. Are these the same factors that workers expecting to retire early perceive as important for the retirement decision, as those who expect to work beyond state pension age? Or the same factors that motivate prolonging work life even further? Knowledge on similarities and differences between these two groups of workers may inform targeted interventions to prevent early retirement as well as interventions to motivate workers, already working beyond the retirement age, to prolong their labour market engagement even further. Therefore, using survey data from a large representative sample of workers aged 55 or older, the present study analyses and compares the self-reported salience of the factors for conditioning retirement intentions presented above, among three different retirement groups: workers expecting to retire before, at, or after the state pension age, respectively. Further, in the same way, the study also analyses and compares factors that could potentially change such retirement decisions and prolong working life.

## 2. Materials and Method

### 2.1. Design

This study is a part of the SeniorWorkingLife project that investigates Push and Stay mechanisms for labour market participation among workers aged 50+ years in Denmark. The study is registered as a cohort study in ClinicalTrials.gov (identifier: NCT03634410) and the open-access protocol is published elsewhere [22]. In the present article, we present cross-sectional data from the baseline questionnaire survey that was sent out in July 2018 and terminated in October 2018. 

### 2.2. Participants

For the present study, a total of 30,000 Danes aged 50 years or more (18,000 employed, 7000 unemployed, 3000 on voluntary early retirement, 2000 on disability pension) were drawn as a probability sample within each specified strata by Statistics Denmark and invited with a personal questionnaire-link via e-Boks (online digital mailbox linked to the Danish social security number) to participate. We only include employed individuals in the current article. Among those who were employed, the response rate to the entire questionnaire was 56%, but for the present analyses, those replying only partly were included as well, yielding a total sample size of 12,269 employed individuals. 

For the purpose of the present article, we divided the sample into three retirement groups (before (n = 6101), at (n = 3766), and after (n = 2402) the state pension age) based on their answer to the question “at which age do you expect to leave the labour market permanently”? Those who expected to retire at least one year before and after, respectively, the official state pension age were defined as ‘before’ and ‘after’ the state pension age. Those expecting to retire within one year before or after were defined as ‘at’ the state pension age.

### 2.3. The Questionnaire

The questions were inspired by The Danish Longitudinal Study of Ageing [23] and the questionnaire is described in more detail in Andersen, Jensen [3]. In short, the question regarding factors conditioning retirement intentions contained 15 multiple-choice response options, provided in random order and shown in Table 2. For possible reasons to stay longer, 15 multiple-choice response options were likewise provided in random order for each respondent and are shown in Table 3. For both questionnaire batteries, the option ‘none of the above’ was provided at the bottom of the multiple-choice questions as the 16th option.

### 2.4. Statistical Analyses

The SurveyFreq procedure of SAS (version 9.4) was used to estimate prevalence and 95% confidence intervals. For the different questions, the SurveyLogistic procedure was used to estimate odds ratios (ORs) and 95% confidence intervals for those intending to leave before and after state pension age, respectively, compared to those leaving at state pension age. The analyses were controlled for sex, age, and highest obtained education. Model-assisted weights were included in the procedures, based on information from high-quality national registers at Statistics Denmark, and took into account sex, age, occupational industry, highest completed education, family income, family type, and origin [22].

## 3. Results

As shown in Table 1, the proportion of men is notably larger among participants expecting to retire after the state pension age, whereas the gender distribution is more even in the other two retirement groups (i.e., before state pension age and at state pension age). The mean age of the participants is highest among those expecting to retire after followed by at the state pension age. The proportion rating their health as bad or not so good is slightly larger among those expecting to retire early and slightly smaller among those expecting to retire after the state pension age. The self-rated physical and psychological strain at work is less among those expecting to retire after the state pension age, and finally, the self-rated work ability is slightly better among those expecting to retire after the state pension age. Table 1 shows the descriptive characteristics of the participants.

### 3.1. Factors Conditioning the Retirement Decision

Table 2 shows prevalence and ORs of expected reasons for leaving the labour market among the three retirement groups. Overall, it seems that the factors that appear to be most relevant are similar across the three retirement groups (Table 2). The factors the largest proportion of the participants pointed towards relate to leisure: the wish to determine for themselves what they want to do, and to have more time for hobbies. The factors the second largest proportion of the participants pointed towards relate to economy and retirement considerations: the possibility of receiving early retirement pension and pension. Finally, not being capable of doing their job is likewise a factor pointed towards by a larger proportion of the participants, followed by economic considerations and poor physical health. 

Comparing participants expecting to retire at the state pension age with those who expect to retire before and after this age, respectively, the results show that those expecting to retire early were a bit, but significantly, more likely to point towards the two factors related to leisure as conditioning their decision to retire (OR: 1.26 (1.14–1.39), 1.14 (1.03–1.26)).

Looking at health, work demands and well-being, there are no significant differences between participants expecting to retire at the state pension age and those expecting to retire early or late, respectively. 

Regarding economy and retirement considerations, not surprisingly, participants expecting to retire early were much more likely to point towards the possibility of receiving voluntary early retirement pension. Likewise, participants expecting to retire at the state pension age were more likely to point towards the possibility of receiving state pension than both of the other retirement groups. In addition, the participants expecting to retire early were less likely than participants expecting to retire at the state pension age to point at economic considerations as conditioning their decision to retire (OR: 0.71 (0.62–0.80)).

When looking at norms, those expecting to retire at the state pension age were more likely than both of the other groups, to point towards the factor that it is common to retire at that age as conditioning their decision to retire.

Although, in general, only a small proportion of the participants pointed towards external factors as expected reasons for retiring, when compared with participants expecting to retire at the state pension age, participants expecting to retire later were *more* likely to point towards the three external factors (OR: 1.53 (1.21–1.94), 1.97 (1.52–2.57), 1.84 (1.31–2.60)), and those expecting to retire earlier were *less* likely to point towards the external factors except for “wish from spouse” (OR: 0.64 (0.50–0.83), 0.50 (0.36–0.71). 

Finally, the participants expecting to retire after the state pension age were more likely than those expecting to retire at the state pension age, to choose the ”none of the above” factors as conditioning their decision to retire (OR: 3.74 (2.75–5.08)).

### 3.2. Possible Reasons for Prolonging Work Participation

Table 3 shows prevalence and ORs of possible reasons for staying longer in the labour market among the three retirement groups. When looking at the factors that would potentially contribute to the participants staying longer in the labour market, the overall pattern of which factors appear to be most important is again similar across the three retirement groups (Table 3). The three factors the largest proportion of the participants point towards are leisure, flexibility, and economy. So particularly more senior days (extra days off for senior employees), the working time being better organised according to their needs, and that it would pay better off economically to continue working, appear to be the most relevant factors motivating the participants to stay at work.

When comparing the retirement groups, the results show that those expecting to retire later were less likely to point towards the two factors related to leisure as possible reasons for them staying in the labour market for longer (OR: 0.59 (0.51–0.68), 0.69 (0.59–0.80)). Regarding flexibility, participants expecting to retire at the state pension age were more likely to point towards “if the working time was better organised according to your needs” than both of the other retirement groups. 

When looking at health and work demands and comparing with participants expecting to retire at the state pension age. Participants expecting to retire early were *more* likely to choose the factors “if your health had been better” (OR: 1.35 (1.17–1.56)) and “if the work was less mentally strenuous” (OR: 1.30 (1.13–1.50)), while those expecting to retire later were *less* likely to point towards the work being less both physically and mentally strenuous as possible reasons for them staying in the labour market longer (OR: 0.55 (0.45–0.69), 0.59 (0.47–0.73)).

Regarding recognition and influence at work, participants expecting to retire at the state pension age were significantly *more* likely to point towards “if the management wanted you to stay longer” than those expecting to retire early and significantly *less* likely than those expecting to retire later. Moreover, those expecting to retire early were a bit, but significantly, more likely to point towards “if your work was appreciated to a greater extent” than those expecting to retire at the state pension age. 

Looking at changes and challenges at work and comparing with participants expecting to retire at the state pension age, results show that participants expecting to retire early were a bit, but significantly, *more* likely to point towards “if there were less demands for adaptation and change” (OR: 1.18 (1.01–1.39)), while participants expecting to retire later, were *less* likely to point at this factor (OR: 0.62 (0.49–0.79)). In addition, those expecting to retire later, were more likely to point at “if there were greater professional challenges” as possible reason for them wanting to stay longer in the labour market (OR: 1.80 (1.36–2.39)).

Regarding the factors related to education, there were no significant differences between the retirement groups.

When looking at the results regarding external factors, participants expecting to retire early were a little, but significantly, *less* likely (OR: 0.76 (0.61–0.95)) to point towards “if there was support from a spouse” and those expecting to retire later were *more* likely (OR: 1.63 (1.29–2.06)) to point towards this factor, than participants expecting to retire at the state pension age.

Finally, participants, expecting to retire after the state pension age, were more likely than participants, expecting to retire at the state pension age, to choose the “none of the above” factors as possible reasons for them wanting to stay longer in the labour market (OR: 1.47 (1.26–1.72)).

## 4. Discussion

The aim of the study was to explore factors conditioning retirement intentions and factors that could potentially prolong working life among workers expecting to retire before, at, and after the state pension age. In general, the results indicate that there are more similarities than differences between these three retirement groups. For all three groups, factors related to leisure, health, and economy were the most salient expected reasons for leaving the labour market and factors related to leisure, flexibility, and economy the most salient possible reasons for prolonging work participation. However, the results also found some differences between the groups. Those expecting to retire before the state pension age were more likely to point towards the desire for more leisure time as the expected reason for retiring, and less likely to point towards if it would pay off better as a possible reason for continuing working. At the same time, they were also more likely to point towards if their health had been better and if their work was less mentally strenuous as possible reasons for prolonging work engagement. Those expecting to retire after the state pension age were more likely to point towards external factors as influencing their retirement decisions.

The most common factors conditioning the retirement decision (independent of retirement group) were related to more leisure time (i.e., to determine what to do and more time for hobbies). When comparing the three groups, results show that participants expecting to retire early were a bit more likely to point towards leisure time as an expected reason for retiring. These findings are in line with previous literature showing that the desire for more leisure time is a motivation for early retirement [14]. Furthermore, the results show that participants, expecting to retire after the state pension age, were less likely to point at desire for more time for hobbies as an expected reason for them retiring, indicating that this is a weaker incentive for this group of older workers. This could reflect, that this group experiences a higher level of work–life balance as also indicated by the results showing that they were less likely to point at more senior days and longer vacations as an incentive to stay in the labour market longer.

Poor physical health was a common factor across the three retirement groups as an expected reason for retiring, which is in line with previous literature [4,5,6,7,8,9,10,11,12]. There were no significant differences between the three retirement groups regarding the role of poor mental and physical health for them retiring, indicating that health problems are a salient factor contributing to retirement for both early and late retirees. These findings provide further support for the existing research reporting associations between poor health and early retirement [1] and findings from qualitative studies showing that employees working beyond retirement age mention poor health as the only reason they would stop working [12]. However, when looking at possible reasons for working longer, participants expecting to retire early were more likely to point at “if my health was better”, which probably reflects that a larger proportion of these participants have health problems.

A large proportion of both workers expecting to retire before and at the age reported the possibility of receiving pension/early retirement pension as an expected reason for their decision to retire. This could reflect that this is a prerequisite to retire at all. Looking at possible reasons to prolong work life, “if it would pay off better economically” was one of the factors the largest proportion of the participants in all three retirement groups pointed at, supporting previous research indicating financial gain as an incentive to continue working [8,10,11]. The participants expecting to retire before the state pension age were, however, less likely to point at this factor, indicating that other factors may play a larger role for this group of workers. Economic incentives can be regulated, e.g., by political reforms, and it would be relevant to investigate the societal cost–benefit of stimulating people to work longer by the use of economic incentives. 

“Not being capable of doing your job” was also one of the factors the largest proportion of the participants in all three retirement groups expect to contribute to the decision to retire. There were no significant differences between the groups indicating that this is equally salient for all three groups. However, when looking at the factors “the work was less physically/mentally strenuous” as possible reasons for staying at work longer, participants expecting to retire later, were less likely to point at these. They also rated both the physical and psychological strain at work as lower indicating that they to a lesser extent have strenuous jobs. Physical and mental demands have been associated with early retirement [2,13,14], it is thus likely that the less strenuous work contributes to this group of the participants expecting to work beyond retirement age. 

Although only a minority point at this, greater professional challenges appear to be a greater incentive to prolong working even further among those expecting to retire after the state pension age. These findings provide further support for research indicating that skill development and challenge motivate prolonging work life [10,11,12,18], but this may apply more to those already expecting to work for longer. However, when asked about opportunities for continuing education and paid education to carry out another job, there were no differences between the three retirement groups and again, only a minority pointing at this as an incentive to prolong working life.

Only a minority of the participants pointed at external factors such as “request from the workplace”, nevertheless, it appears to play a larger role in the decision to retire among employees planning to retire after the state pension age. This is further supported by the results that they were more likely to point at “if the management wanted you to stay longer” and “if there was support from my spouse”, as possible reasons for them staying longer, in other words, they are willing to continue working if encouraged to do so. 

### Strengths and Weaknesses of the Study

The strengths of the study include the careful procedure to ensure that the estimates are representative of workers in Denmark aged 50 years or older. To produce the large representative sample used in the study, Statistics Denmark drew a probability sample among all eligible Danish residents age 50 years or older and combined this with model-assisted weights based on high-quality national registers. Furthermore, the electronic questionnaire was designed to reduce the risk of bias by randomising the order of the response options.

The questions analysed in the present study were of a factual nature, i.e., the participants were asked to choose which of the listed factors they believed would contribute to their decision to retire as well as which factors they believed might motivate them to stay at work for longer. The listed factors were inspired from the literature. For these reasons, it is not relevant to validate the questionnaire. Nevertheless, one could argue that it is a limitation that we did not include an exhaustive list of possible factors, conditioning the retirement decision, and thus may have missed out potential important factors. However, firstly, as the list of factors included in the questionnaire was inspired from the rather extensive literature on the topic, we do believe that we have included the most important factors, secondly, we had to limit the number of factors included in the questionnaire to keep it at a reasonable length.

A weakness of the study is that the results are based on cross-sectional data and intended behaviour and, therefore, do not reveal whether the identified factors relate to actual behaviour (i.e., the actual time-point of retirement). Nevertheless, the purpose of the study was to investigate similarities and differences between the three retirement groups on factors they perceived as important for their retirement decisions. The data are therefore adequate for the purpose of the study. 

In the present study, we estimated ORs. However, it can be argued that the ORs overestimate the values found. Thus, a direct comparison with other studies using, e.g., prevalence ratios cannot be done. For interpretation of the present results, it is therefore also important to consider the respective prevalence (percentages in the first three columns of Table 2 and Table 3), and not only the ORs.

Finally, the applicability of the results to countries applying different arrangements of retirement benefits and labour market protection needs to be investigated in future research. For example, van der Wel, Dahl [24] conclude that the welfare system of Scandinavian countries is better at protecting against non-employment due to illness than other systems. The generalisability of the present study, therefore, may only apply to workers in welfare states.

## 5. Conclusions

All in all, the results indicate that the factors, that appear to be most salient when making the decision to retire, overall are similar for employees expecting to retire before, at, and after the state pension age, namely, leisure time, capability to do the job, health, and economy. Thus, initiatives to help ensure better work–life balance, enhance demand and resource fit, supporting healthy lifestyle, and that it pays off economically to continue working may help prevent early retirement and motivate prolonging work life beyond retirement age. However, the results also indicate that ensuring work–life balance and finding ways to make the work less strenuous are particularly relevant to prevent early retirement. The participants expecting to retire after the state pension age have already been prevented from leaving work early, so to say. To motivate this group of workers to prolong work life even further, in addition to focussing on work–life balance, healthy lifestyle, and economic incentives to continue working, the results indicate that professional challenges at work and being encouraged to continue working by the spouse and the workplace, may serve as motivation for at least some of these workers. 

## Figures and Tables

**Table 1:** Background information for the three retirement groups.

	Before State Pension Age (N = 5741–6101)	At State Pension Age (N = 3510–3779)	After State Pension Age (N = 2186–2420)
Men (%)	47	53	71
Women (%)	53	47	29
Age (mean)	55.3	56.9	59.7
Self-rated health as bad/not so good (%)	12	10	6
Weekly working hours (mean)	38.4	38.8	39.2
Self-rated physical strain at work (mean/scale 0–10)	3.7	3.4	2.8
Self-rated psychological strain at work (mean/scale 0–10)	5.6	5.5	4.9
Self-rated workability (mean/scale 0–10)	7.8	8.0	8.2

N Varies due to Omitted Responses.

**Table 2:** Factors conditioning the retirement decision.

				OR (95% CI) *	
You Wrote That You Expect to Leave the Labour Market at Age XX. Please Choose the Factors That May Play a Role in This.	Before State Pension Age (N = 6101) (%)	At State Pension Age (N = 3766) (%)	After State Pension Age (N = 2402) (%)	Before vs. at State Pension Age	After vs. at State Pension Age
Leisure					
That you want do determine yourself what you want to do	50	46	46	**1.26** (1.14–1.39)	0.93 (0.81–1.06)
To have more time for hobbies	47	44	32	**1.14** (1.03–1.26)	**0.57** (0.49–0.65)
Health, work demands, and well-being					
That you will not be capable of doing your job	25	25	24	0.92 (0.82–1.03)	1.17 (1.00–1.36)
Poor physical health	19	17	16	1.07 (0.93–1.22)	1.15 (0.96–1.36)
That you do not thrive at the workplace	8	8	9	0.96 (0.80–1.15)	1.14 (0.90–1.43)
Poor mental health	4	5	6	0.79 (0.63–1.0)	1.20 (0.91–1.58)
Economy and retirement considerations					
Possibility of receiving pension	21	46	14	**0.28** (0.25–0.31)	**0.21** (0.18–0.25)
Economic considerations	17	22	23	**0.71** (0.62–0.80)	1.09 (0.94–1.27)
Possibility of receiving voluntary early retirement pension	35	6	1	**10.96** (9.01–13.32)	**0.14** (0.09–0.24)
Good retirement conditions at the workplace	6	5	6	1.12 (0.91–1.38)	1.27 (0.98–1.65)
Norms					
It is common to leave at that age in your type of work	9	15	9	**0.55** (0.47–0.64)	**0.64** (0.52–0.78)
To make space for younger employees	12	10	9	1.13 (0.97–1.33)	0.86 (0.69–1.08)
External factors					
Wish from spouse	6	6	9	1.18 (0.96–1.44)	**1.53** (1.21–1.94)
Termination of employment	3	4	8	**0.64** (0.50–0.83)	**1.97** (1.52–2.57)
At the request of the workplace	1	3	5	**0.50** (0.36–0.71)	**1.84** (1.31–2.60)
None of the above	1	3	9	**0.55** (0.39–0.79)	**3.74** (2.75–5.08)

OR: odds ratio; CI: confidence interval. Statistical significant OR’s are highlighted in **bold** * Controlled for sex, age, and educational group.

**Table 3:** Possible reasons for prolonging work participation.

				OR (95% CI) *	
You Wrote That You Expect to Leave the Labour Market at Age XX. Please Choose the Factors That Would Make You Stay in the Labour Market for Longer.	Before State Pension Age (N = 6101) (%)	At State Pension Age (N = 3750) (%)	After State Pension Age (N = 2392) (%)	Before Vs. at State Pension Age	After Vs. at State Pension Age
**Leisure**					
If there were more senior days	44	43	28	0.99 (0.89–1.09)	**0.59** (0.51–0.68)
If there was a possibility for longer vacations	30	30	21	0.97 (0.87–1.08)	**0.69** (0.59–0.80)
**Flexibility**					
If the working time was better organised according to your needs	34	37	28	**0.84** (0.75–0.93)	**0.79** (0.68–0.91)
**Economy**					
If it would pay better off economically	26	33	29	**0.71** (0.64–0.79)	0.89 (0.77–1.02)
**Health and work demands**					
If your health had been better	17	13	12	**1.35** (1.17–1.56)	0.94 (0.77–1.16)
If the work was less physically strenuous	19	16	8	1.13 (0.98–1.29)	**0.55** (0.45–0.69)
If the work was less mentally strenuous	17	14	8	**1.30** (1.13–1.50)	**0.59** (0.47–0.73)
**Recognition and influence at work**					
If the management wanted you to stay longer	11	16	22	**0.59** (0.51–0.68)	**1.39** (1.18–1.63)
If your work was appreciated to a greater extent	13	11	9	**1.21** (1.04–1.41)	0.93 (0.75–1.15)
If you got more influence on planning the work	11	12	11	0.91 (0.78–1.06)	1.04 (0.85–1.28)
**Changes and challenges at work**					
If there were less demands for adaptation and change	12	11	6	**1.18** (1.01–1.39)	**0.62** (0.49–0.79)
If there were greater professional challenges	4	4	7	0.91 (0.71–1.17)	**1.80** (1.36–2.39)
**Education**					
If your opportunities for continuing education were better	5	5	5	0.88 (0.71–1.11)	1.17 (0.87–1.58)
If you got a paid educational course to carry out another job (not necessarily at the same workplace)	6	6	5	0.88 (0.72–1.09)	0.87 (0.65–1.16)
**External factors**					
If there was support from spouse/cohabitant/partner	4	6	10	**0.76** (0.61–0.95)	**1.63** (1.29–2.06)
**None of the above**	18	19	26	0.94 (0.82–1.06)	**1.47** (1.26–1.72)

* Controlled for sex, age, and educational group. Statistical significant OR’s are highlighted in **bold.**

## Data Availability

The authors encourage collaboration and use of the data by other researchers. Data are stored on the server of Statistics Denmark, and researchers interested in using the data for scientific purposes should contact the project leader L.L.A., lla@nfa.dk.
